# Thyroid dysfunction and osteoporosis: A two-sample bidirectional Mendelian randomization study

**DOI:** 10.1097/MD.0000000000044779

**Published:** 2025-09-26

**Authors:** Minshun Zhu, Yuwen Wang, Yu Liang, Xingyu Wang, Jianhua Zhang, Dongyan Wang, Mao Yang

**Affiliations:** a The First Affiliated Hospital of Anhui University of Chinese Medicine, Hefei, China; b Anhui University of Traditional Chinese Medicine, Hefei, China; c Lianyungang Hospital Affiliated to Xuzhou Medical University, Lianyungang, China.

**Keywords:** causal inference, hyperthyroidism, hypothyroidism, mendelian randomization, osteoporosis

## Abstract

Previous observational studies have suggested a potential link between hyperthyroidism and osteoporosis (OP) risk. However, the relationship between hypothyroidism and osteoporosis risk remains uncertain due to the inherent limitations of observational studies, such as reverse causality and confounders, which complicate the determination of causality. To address these issues, we conducted a 2-way, 2-sample Mendelian randomized study using instrumental variables selected from a large genome-wide association study meta-analysis dataset of European origin. This approach allowed us to assess the causal effects of hyperthyroidism and hypothyroidism on OP. Additionally, inverse Mendelian randomization (MR) analysis was employed to estimate the causal effect of OP on hyperthyroidism and hypothyroidism. The primary analytical method used was inverse variance weighting, supplemented by MR-Egger and weighted median methods. Heterogeneity tests, sensitivity analyses, and assessments for horizontal pleiotropy complemented the primary analysis. The MR analysis revealed a causal relationship between hyperthyroidism (odds ratio [OR] = 1.17, 95% confidence interval [CI] 1.00–1.36; *P* = 3.61E‐07) and hypothyroidism (OR = 1.01, 95% CI 1.00–1.03; *P* = .013) and the risk of OP. Conversely, no causal relationship was found between OP and hyperthyroidism (OR = 1.00, 95% CI 0.98–1.09; *P* = .208) or hypothyroidism (OR = 1.11, 95% CI 0.92–1.33; *P* = .297) in reverse MR analysis. Therefore, our study confirms a causal link between hyperthyroidism and hypothyroidism with OP, suggesting that individuals with these conditions may have an increased risk of developing osteoporosis. Further research is needed to elucidate the underlying biological mechanisms and provide robust evidence for clinical guidelines aimed at preventing OP.

## 1. Introduction

Osteoporosis (OP) is a bone disease characterized by reduced bone mineral density (BMD), impaired microarchitecture, and imbalances in bone formation and resorption, leading to an increased risk of fragility fractures in patients.^[1]^ The number and prevalence of the disease are increasing every year. In 2013, 22 million women and 5.5 million men in the European Union countries were known to have OP, resulting in 3.5 million fragility fractures per year, with 30% of women and 20% of men over 50 years of age experiencing a fracture.^[2]^ The higher prevalence in women than in men may be due to the postmenopausal decline in estrogen levels, lower peak bone mass in women than in men, and different patterns of bone accumulation and loss in women than in men.^[3,4]^ The global prevalence of OP in people aged 50–59, 60–69, and 70–79 years is 11.4%, 24.8%, and 37.6%, respectively.^[5]^ The pathogenesis of OP is complex, involving a variety of clinical, nutritional, behavioral, medical, and genetic factors.^[6–8]^ Currently, studies have demonstrated that abnormal thyroid hormone secretion plays a crucial role in the development of OP.^[7,9]^ Calcitonin, a thyroid-secreted peptide hormone, regulates bone resorption by inhibiting resorption and calcium release, thereby reducing blood calcium levels. It also contributes to OP development indirectly by stimulating osteoblasts to secrete more sclerostin.^[10]^

The thyroid gland is the largest endocrine gland in the body and is responsible for synthesizing and secreting thyroid hormones. Hyperthyroidism is a type of thyroid dysfunction, a condition caused by an overactive thyroid gland leading to excessive production and secretion of thyroid hormones. If left untreated, it can lead to severe OP and pathologic fractures. The most common causes of hyperthyroidism are Graves’ disease, toxic multinodular goiter, and toxic adenomas.^[11]^ Hyperthyroidism accelerates bone resorption and formation, shortening the bone resorption period by 60% and the bone formation period by about 30%. This shortens the bone remodeling cycle and increases its frequency of initiation. In addition, the increase in bone formation is less than the increase in bone resorption, resulting in a decrease in bone mass of about 10% per cycle.^[12]^ A decrease in BMD of about 12% to 20% also occurs, and there is little or no increase in BMD after treatment. This is coupled with decreased calcium absorption and increased loss, resulting in a negative calcium balance. Simultaneously, renal 1-alpha-hydroxylase activity is reduced, leading to a decrease in osteotriol synthesis. Consequently, impaired calcium absorption in the gastrointestinal tract worsens the negative calcium balance, accelerating the progression of OP.^[13–15]^

Hypothyroidism, another form of thyroid dysfunction, is caused by insufficient synthesis or production of thyroid hormones. Autoimmune damage, thyroid surgery, and medications are the main causes of its development.^[16]^ The overall prevalence of the disease and the prevalence of untreated hypothyroidism are increasing every year.^[17]^ Whether hypothyroidism causes osteoporosis is controversial. In a 1-year observational study, researchers compared 40 female patients with hypothyroidism to 41 healthy women and found no statistically significant differences in femoral neck and lumbar spine bone density between the 2 groups.^[18]^ However, another observational study found that 72% of patients with hypothyroidism were also diagnosed with bone loss or OP.^[19]^ Additionally, the study revealed that the bone metabolic cycle is shortened, the bone remodeling cycle is prolonged, and the risk of fracture is increased in hypothyroidism.^[13,20]^ All of these findings suggest a strong relationship between hypothyroidism and OP. However, variations in the results of these studies may be influenced by factors such as gender, sample size, and age. Nonetheless, there is currently no clear evidence indicating that hypothyroidism increases the risk of OP and fractures.^[9]^

Prospective randomized controlled trials are often considered the gold standard in inferring epidemiological causality. However, they come with drawbacks such as medical ethical constraints, expense, and time consumption. Moreover, observational studies face challenges in avoiding biases from confounding factors and reverse causation. Therefore, accurately inferring a causal relationship between hypothyroidism and OP proves difficult. Mendelian randomization (MR) offers a novel method using genetic variations for epidemiological causal inference.^[21]^ It avoids confounders and infers causality because alleles exposing genetic variants are randomly assigned.^[22]^ Therefore, this study employed MR to explore the potential pathophysiology or causality of hyperthyroidism and hypothyroidism on OP, as well as risk factors. Additionally, we examined the direction of causality by reversing exposure and outcome.

## 2. Methods

### 2.1. Study design and instrument selection

**Figure 1.**
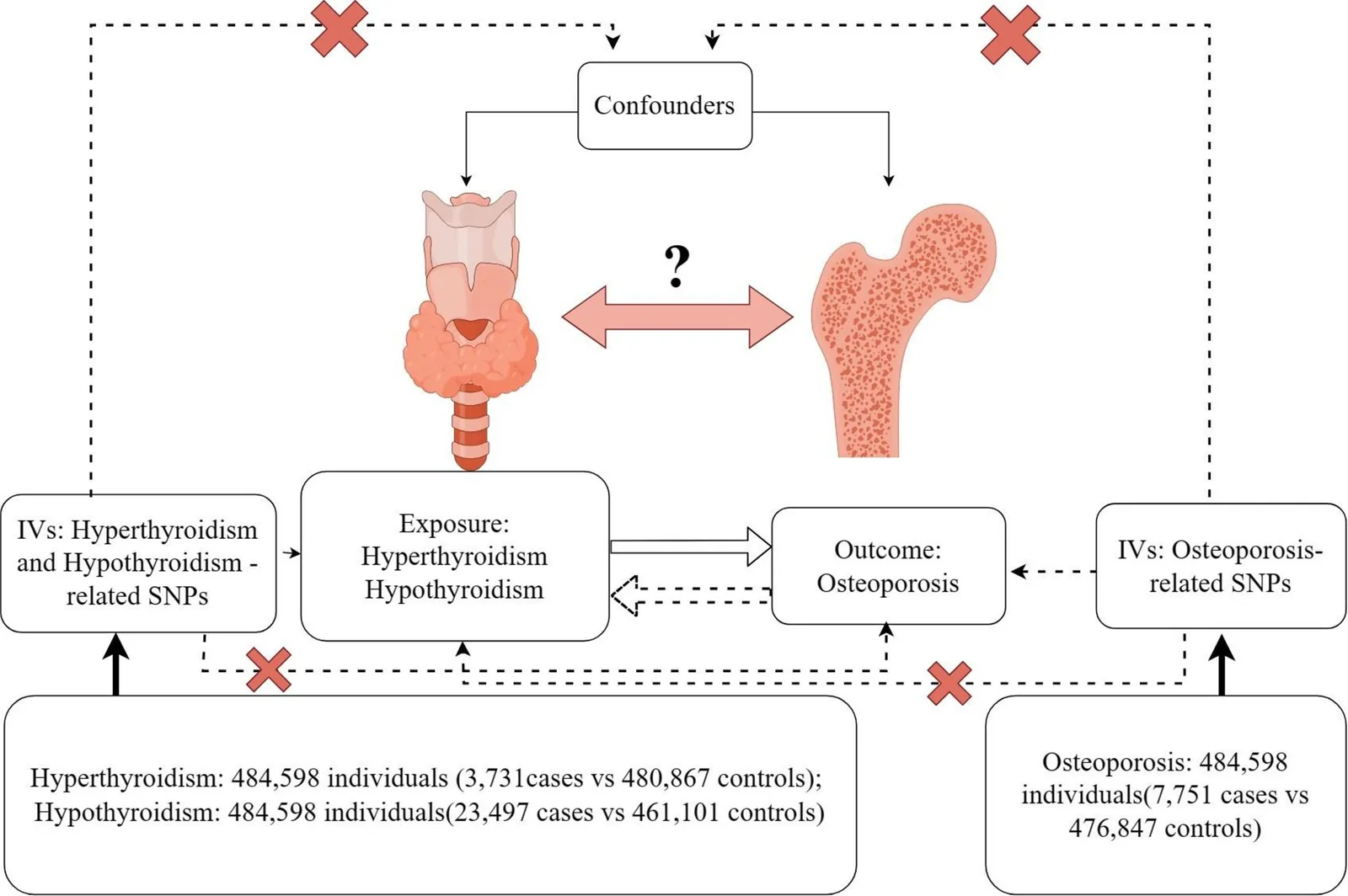
The designs and assumptions for the bidirectional MR study of the causal association of thyroid dysfunction disease and OP risk. MR = Mendelian randomization, OP = osteoporosis.

A 2-sample MR study was conducted to identify causal relationships. The study utilized the 2-sample MR package from the R package (version 4.2.3, R Foundation for Statistical Computing, Vienna, Austria). MR analysis was conducted according to 3 hypotheses: Selected single nucleotide polymorphisms (SNPs) should be strongly associated with exposure. Selected SNPs should not be associated with the outcome through confounders. Selected SNPs should influence the outcome through exposure but not be directly associated (refer to Fig. 1). All information in this study was obtained from public databases or existing publications and did not require additional ethical approval.

Regarding instrumental variables (IVs), we selected SNPs that exhibited significant exposure correlation (*P* < 5 × 10^‐8^). *F* and *r*^2^ values were calculated to ensure the validity of each SNP. In our study, linkage disequilibrium was eliminated (*r*^2^ < 0.01, 5000 kb). Additionally, all SNPs with palindromic structures and SNPs with *F* values < 10 were removed.^[23]^

### 2.2. Data sources

All genome-wide association study (GWAS) data were obtained from the integrative epidemiology unit website (https://gwas.mrcieu.ac.uk/). GWAS data for both exposures and outcomes were sourced from the European Bioinformatics Institute GWAS catalogue (https://www.ebi.ac.uk/). Hyperthyroidism (ID: ebi-a-GCST90038636) and hypothyroidism (ID: ebi-a-GCST90038637) were selected as exposures, while OP (ID: ebi-a-GCST90038656) was chosen as the outcome. All disease study samples were derived from European populations, comprising both males and females. Summary information on exposures and outcomes is presented in Table 1.

**Table 1:** Summary information on exposures and outcomes.

Exposure/outcome	Database	Ancestry	Year	Cases	Controls	Sample size	Number of SNPs
Hyperthyroidism (ebi-a-GCST90038636)	EBI	European	2021	3731	480,867	484,598	9587,836
Hypothyroidism (ebi-a-GCST90038637)	EBI	European	2021	23,497	461,101	484,598	9587,836
Osteoporosis (ebi-a-GCST90038656)	EBI	European	2021	7751	476,847	484,598	9587,836

EBI = European Bioinformatics Institute.

### 2.3. Statistical analysis

Bidirectional MR method was used to estimate the causal relationship between hyperthyroidism and hypothyroidism and OP. MR-Egger method, weighted median (WM), and inverse variance weighting (IVW) methods were used. The main analytical method used to assess causality was the IVW method. Sensitivity analysis involving Cochran *Q* test, MR-Egger regression, and leave-one-out analysis were performed to verify the accuracy of the results we obtained. Cochran *Q* test was used to assess the heterogeneity of SNPs (*P* > .05 indicates no heterogeneity).^[24]^ Genetic diversity that affects the results through alternative pathways is referred to as pleiotropy.^[25]^ Horizontal pleiotropy was subsequently tested using MR-Egger regression, with effect estimates expressed as intercepts.^[26]^ To assess the overall impact of specific genetic variants, sensitivity analyses were conducted employing leave-one-out analysis to evaluate the effect of each SNP on the outcome individually. Additionally, by recalculating the causal estimates, the effect of each unique genetic variant could be determined.^[27]^

## 3. Results

### 3.1. IV selection

Genome-wide significant SNPs loci were screened using R software. Hyperthyroidism as an exposure factor yielded 29 SNPs as IVs; hypothyroidism as an exposure factor yielded 163 SNPs as IVs; and OP as an exposure factor yielded 17 SNPs as IVs (Tables S1–S3, Supplemental Digital Content, https://links.lww.com/MD/Q117).

### 3.2. Causal association of hyperthyroidism and hypothyroidism on OP

**Figure 2.**
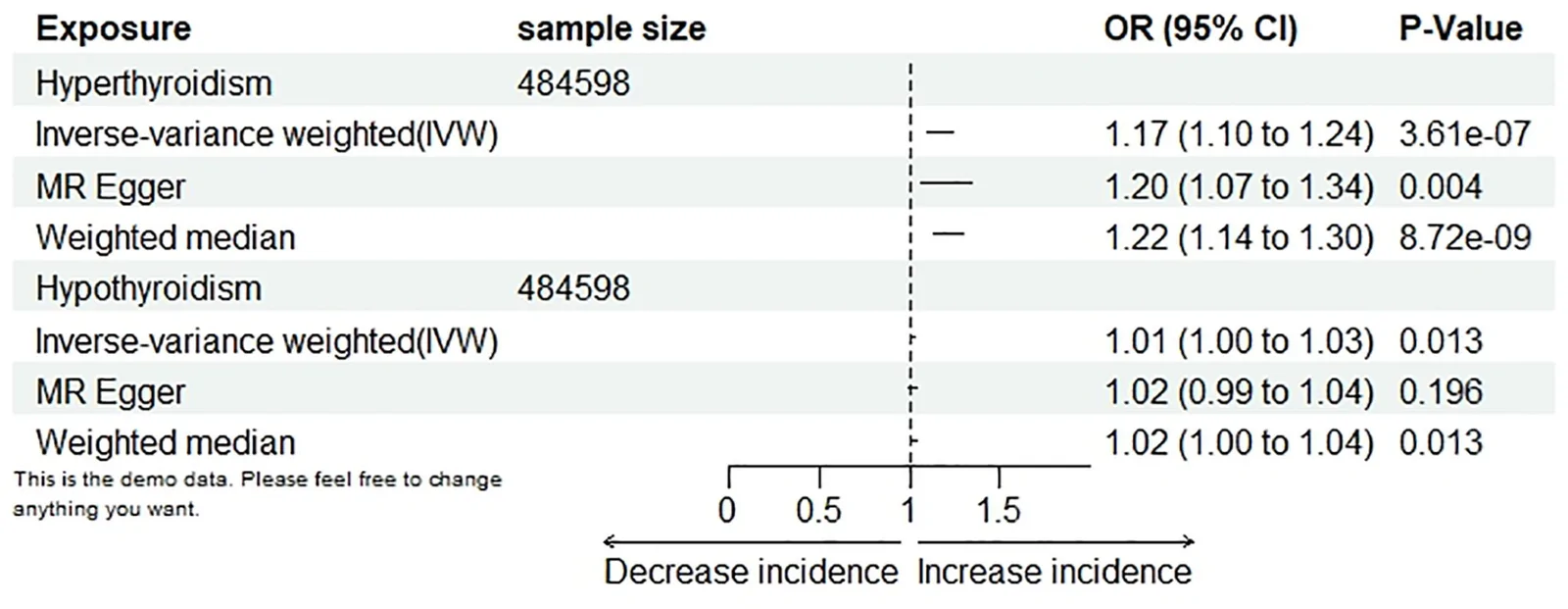
MR results of the causal association between hyperthyroidism and hypothyroidism and OP using 3 methods. MR = Mendelian randomization, OP = osteoporosis.

IVW analysis demonstrated a positive causal association between hyperthyroidism and OP (odds ratio [OR] = 1.17, 95% confidence interval [CI] 1.00–1.03; *P* = 3.61E‐07). Similarly, the MR-Egger method (OR = 1.20, 95% CI 0.99–1.04; *P* = .004) and WM method (OR = 1.22, 95% CI 1.00–1.04; *P* = 5.31E‐09) also detected this correlation. Additionally, IVW analysis revealed a positive causal relationship between hypothyroidism and OP (OR = 1.01, 95% CI 1.00–1.03; *P* = .013), with the WM method (OR = 1.02, 95% CI 1.00–1.04; *P* = .014) also detecting this correlation (Fig. 2).

**Figure 3.**
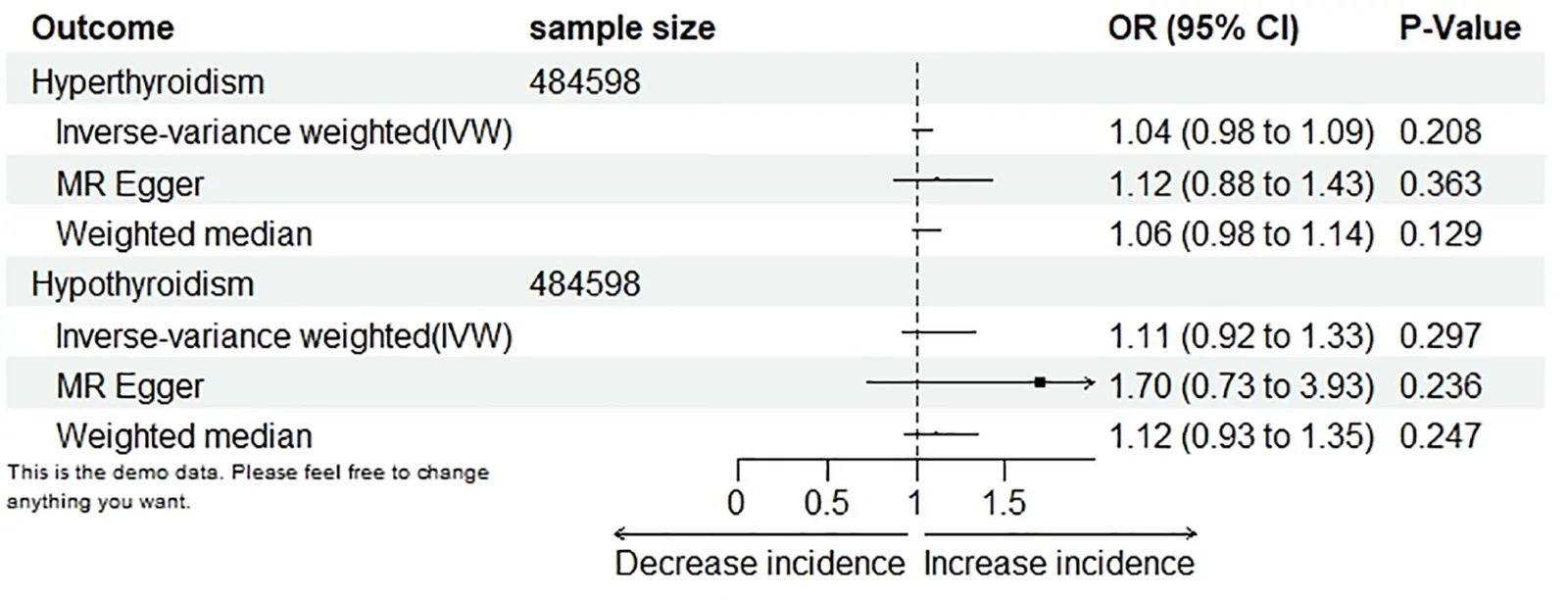
MR results of the causal association between OP and hyperthyroidism and hypothyroidism using 3 methods. OP = osteoporosis.

Additionally, IVW analysis revealed no correlation between OP and the risk of developing hyperthyroidism (OR = 1.00, 95% CI 0.98–1.09; *P* = .208) and hypothyroidism (OR = 1.11, 95% CI 0.92–1.33; *P* = .297) in the reverse MR studies where OP was used as an exposure, indicating no causal relationship between any of them (Fig. 3).

### 3.3. Sensitivity analysis

The results of Cochran *Q* test are presented in Table 2. The analysis revealed some heterogeneity between the SNPs for hyperthyroidism (*Q* = 47.4, *P* = .012) and hypothyroidism (*Q* = 225.9, *P* = .001) and OP. Particularly noteworthy is the Egger-intercept assay, which indicated the absence of multivalency testing at all orientation levels (*P* > .05), thereby enhancing the reliability of causality inferred from our findings.

**Table 2:** The results of Cochran *Q* test.

Exposure	Test for directional horizontal pleiotropy			Cochran *Q* test	
	Egger-intercept	SE	*P*-value	*Q*	*Q*-pval
Hyperthyroidism (ID: ebi-a-GCST90038636)	‐0.000079	0.000156	.617	47.4	.012
Hypothyroidism (ID: ebi-a-GCST90038637)	‐0.000003	0.000058	.954	225.9	.001

OP = osteoporosis.

**Figure 4.**
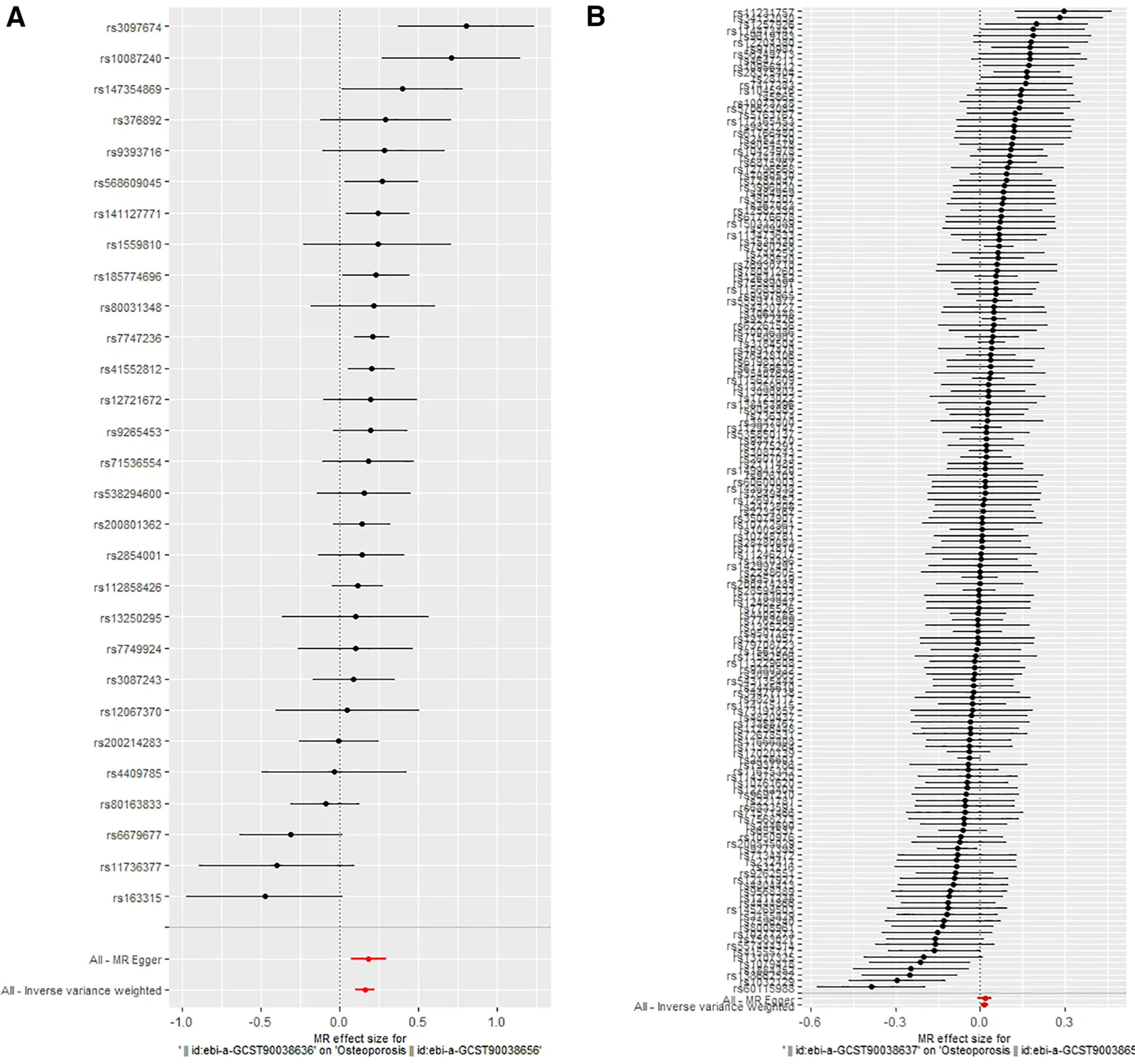
(A) Shows the fixed-effects IVW analysis for the causal association of hyperthyroidism and hypothyroidism with OP. (B) Shows the fixed-effects IVW analysis for the causal association of hyperthyroidism and hypothyroidism with OP. IVW = inverse variance weighted, OP = osteoporosis.

**Figure 5.**
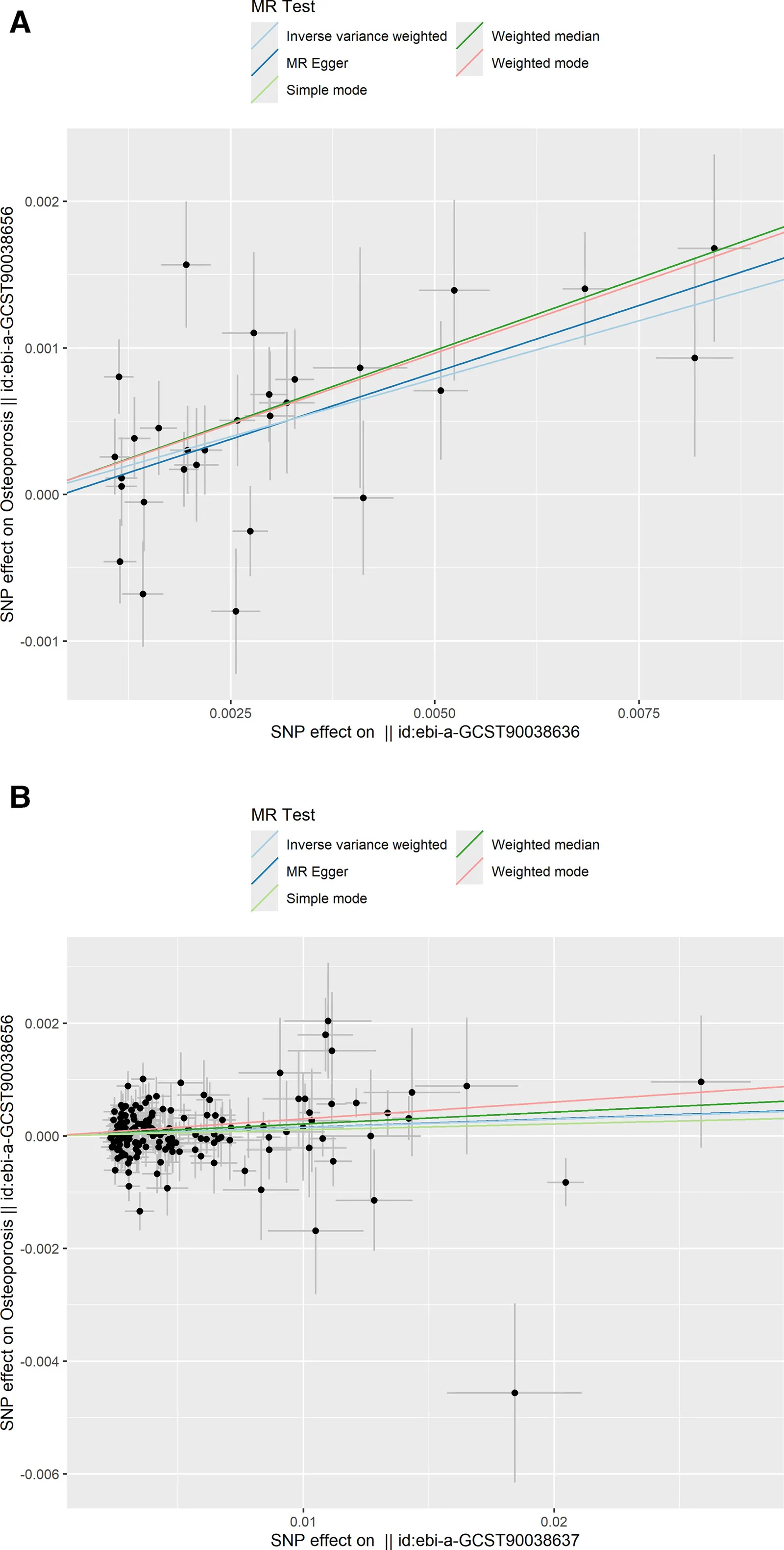
(A) Scatterplot showing the effect of genetic variants on the impact of hyperthyroidism and hypothyroidism on OP. (B) Scatterplot showing the effect of genetic variants on the impact of hyperthyroidism and hypothyroidism on OP.

**Figure 6.**
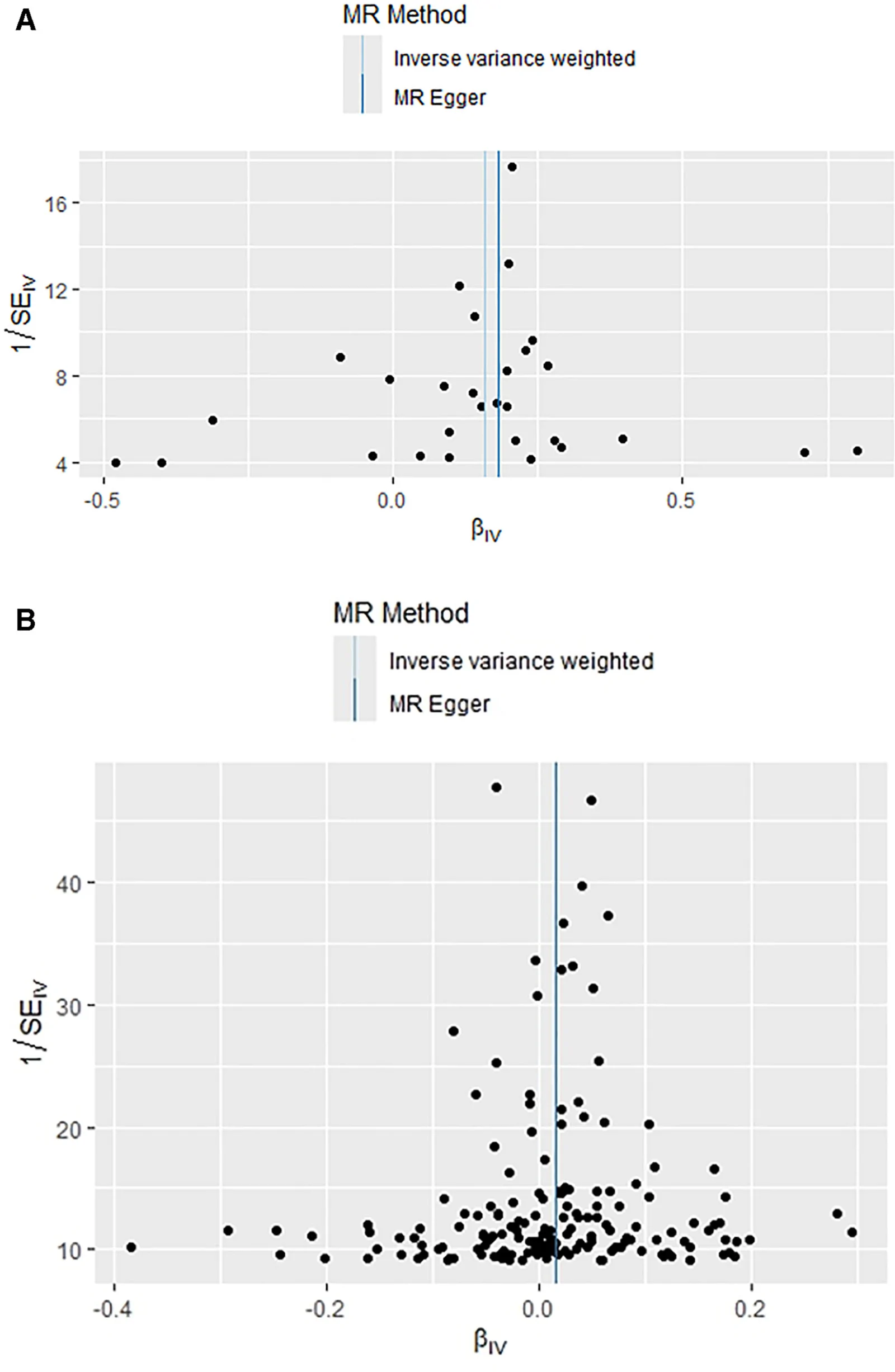
(A) Shows a funnel plot of the causal relationship between hyperthyroidism and hypothyroidism and OP. (B) Shows a funnel plot of the causal relationship between hyperthyroidism and hypothyroidism and OP. OP = osteoporosis.

**Figure 7.**
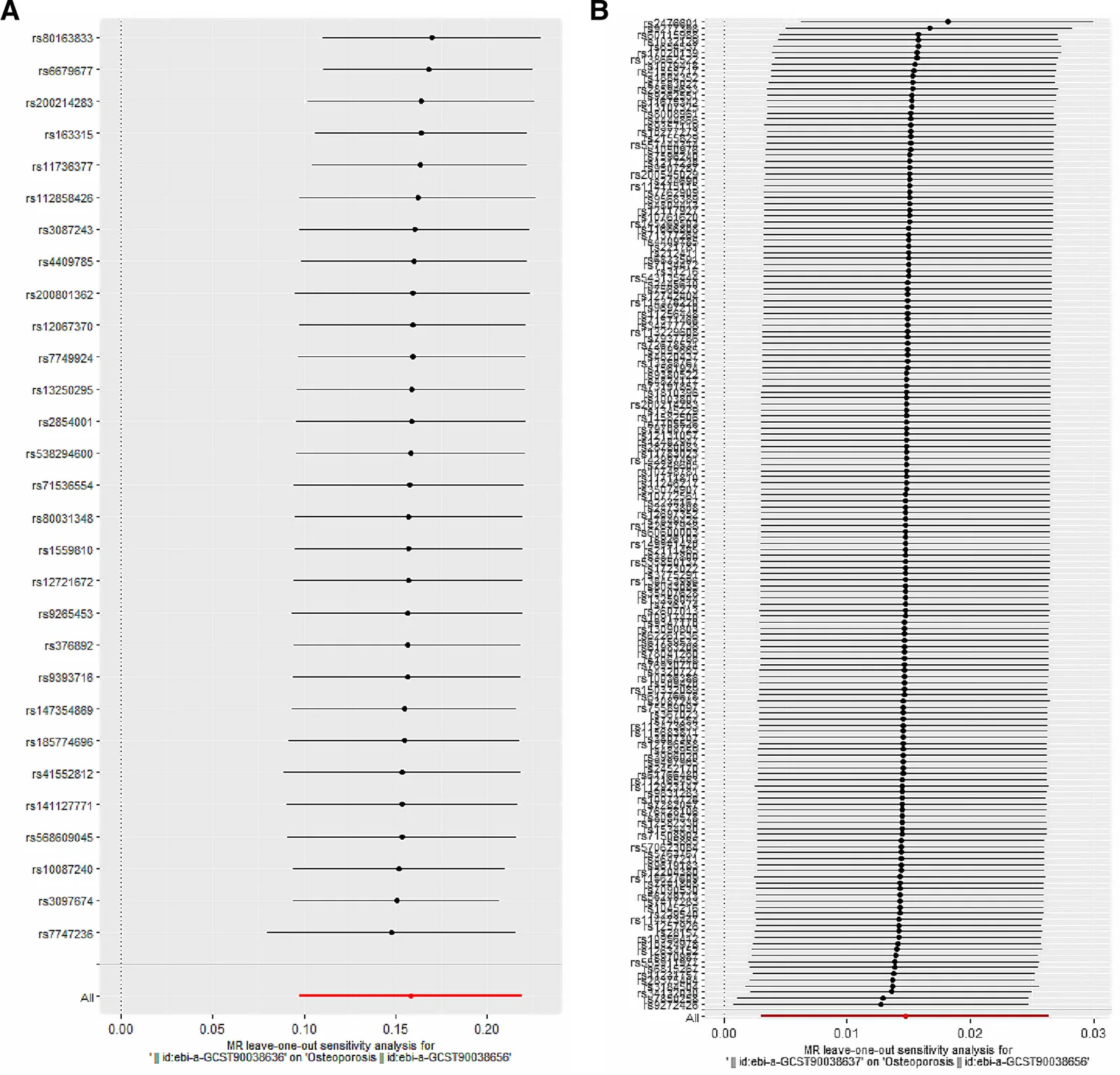
(A) Shows plots of omitted analysis of the causal effects of hyperthyroidism and hypothyroidism with respect to OP. (B) Shows plots of omitted analysis of the causal effects of hyperthyroidism and hypothyroidism with respect to OP. OP = osteoporosis.

In addition, fixed-effects IVW analysis of the causal association of hyperthyroidism and hypothyroidism with OP is presented (Fig. 4). The black dots and bars represent causal estimates and 95% CIs using each SNP. Scatterplots illustrate the effect of genetic variation on hyperthyroidism and hypothyroidism on OP, with the slope of the solid line indicating the magnitude of association estimated from the MR analysis (Fig. 5). The symmetry of the funnel plot confirmed consistent results (Fig. 6). Furthermore, the leave-one-out method demonstrated that the causal effect with OP was not significantly affected by the omission of any individual SNP (Fig. 7). These findings suggest that the results of the causal effect of hyperthyroidism and hypothyroidism with OP are stable and reliable.

## 4. Discussion

The development of OP has long been strongly associated with abnormal thyroid function.^[28,29]^ While current studies have predominantly focused on the impact of hyperthyroidism on the risk of OP development, fewer investigations have explored whether hypothyroidism influences the risk of OP development and whether a causal relationship exists between the 2. The present study is the first to utilize a bidirectional, 2-sample MR method exclusively with large-scale GWAS data to investigate the potential causal relationship between hyperthyroidism, hypothyroidism, and OP. The results of the primary analysis method indicated a potential positive association between both hyperthyroidism and hypothyroidism and OP. Conversely, the inverse MR evidence revealed no potential association between OP and the risk of developing hyperthyroidism and hypothyroidism.

Our MR results support the well-established findings from epidemiological studies regarding hyperthyroidism, indicating its negative impact on bone health, even in the presence of higher-than-normal levels of thyroid-stimulating hormone (TSH).^[30,31]^ A prospective cohort study involving 5458 individuals demonstrated greater bone loss at the femoral neck in the hyperthyroid population compared to the general population. Subsequent studies have also indicated that lower TSH levels are associated with increased risks of hip and bone fractures.^[32]^ Excessive thyroid hormone in childhood prematurely stimulates growth plates and cranial sutures, resulting in short stature and premature closure of cranial sutures.^[33,34]^ In adults, hyperthyroidism accelerates bone turnover, reduces BMD, shortens the bone remodeling cycle, and disrupts the balance between bone formation and resorption^,[5,33,35]^ thereby elevating the risk of fractures.^[5,33,36]^ Normal functioning and release of TSH play a critical role in bone metabolism. Although evidence on the impact of TSH on osteoblast activity is conflicting, TSH is widely regarded as a primary inhibitor of bone turnover.^[37,38]^ In a case-control study conducted in Denmark in 2000, Vestergaard et al found that the risk of any fracture increased within the first 5 years after the diagnosis of hyperthyroidism.^[39]^

Literature review revealed hypothyroidism as a significant factor in fracture occurrence,^[40]^ Vestergaard discovered that within 2 years of hypothyroidism diagnosis, the fracture risk temporarily rose, primarily among individuals over 50 years old, and predominantly involving the forearm,^[41]^ and in another of his studies found that the risk of any fracture was increased within the first 10 years after a diagnosis of hypothyroidism.^[39]^ Similarly, Tuchendler discovered a higher fracture risk among hypothyroid patients.^[37]^ However, a meta-analysis concluded that hypothyroidism did not elevate fracture risk.^[34]^ Additionally, a study indicated that hypothyroidism itself does not cause OP and may be associated with overtreatment.^[18,42]^ The inconsistent conclusions regarding the association between hypothyroidism and OP create confusion for clinicians and impact clinical decision-making. In this study, the potential causal relationship between hypothyroidism and OP was explored at the genetic level through SNP analysis using MR. The findings revealed that hypothyroidism could elevate the risk of OP, potentially linked to disturbances in bone metabolism. This is supported by Kosińska et al discovery of reduced bone metabolism indices, including calcium and serum osteocalcin concentration, alongside systemic and bone metabolism abnormalities associated with hypothyroidism.^[43]^ Furthermore, children with hypothyroidism exhibit a heightened risk of OP, likely attributed to bone metabolism disturbances. This is evidenced by growth retardation, delayed bone age, and endochondral ossification disruptions observed in pediatric hypothyroid patients.^[44]^

Currently, the following features exist in this study. First, for the first time, we identified and demonstrated that there is indeed a potential causal relationship between hypothyroidism and the development of OP by means of MR analysis, and that there is a positive correlation. Second, bidirectional MR analysis ensured that the inference of causality between 2 thyroid diseases (hyperthyroidism and hypothyroidism) and OP was bidirectional and complemented previous observational studies where the MR design strengthened the inference of causality. Third, multiple sensitivity analysis were performed and confidence in the identified causality was strengthened by the finding that estimates from different models were generally consistent. Finally, we used GWAS data from a large sample of European populations for both exposure endpoints to obtain sufficient statistical power to assess potential causality between diseases as well as to reduce confounding bias.

However, this study has several limitations. Firstly, it focused solely on a European population, raising questions about the generalizability of our findings to other populations. Additional data from non-European populations and further investigations are warranted to validate our results. Secondly, we relaxed the selection criteria (*P* < 5 × 10^‐8^, *r*^2^ = 0.01, kb = 5000) due to a limited number of SNPs meeting the inclusion criteria, possibly resulting in false positives. Despite employing the random effects model and conducting multiplicity testing, the study exhibited considerable heterogeneity and residual bias that remained unaddressed. Furthermore, due to incomplete GWAS data on other thyroid diseases, our study was restricted to 2 exposure datasets. Future MR studies can be pursued once data on related diseases become more comprehensive.

## 5. Conclusion

This study uncovers the potential impact of abnormal thyroid hormone secretion on OP from a genetic perspective. While hyperthyroidism aligns with previous research in elevating OP risk, hypothyroidism presents an unexpected association, potentially linked to bone metabolism abnormalities warranting further investigation.

## Acknowledgments

The generous sharing of GWAS summary statistics facilitated this study. We thank the Figdraw platform for providing drawing materials. We are grateful to the participants, researchers and staff involved in the many other studies from which we obtained the data needed for this report.

## Author contributions

**Conceptualization:** Minshun Zhu, Yuwen Wang.

**Data curation:** Minshun Zhu, Yuwen Wang.

**Formal analysis:** Yu Liang.

**Investigation:** Xingyu Wang.

**Methodology:** Jianhua Zhang.

**Supervision:** Jianhua Zhang.

**Visualization:** Dongyan Wang, Mao Yang.

**Writing – original draft:** Dongyan Wang, Mao Yang.

**Writing – review & editing:** Minshun Zhu.

## Supplementary Material

## References

[1] Styrkarsdottir U Thorleifsson G Gudjonsson SA. Sequence variants in the PTCH1 gene associate with spine bone mineral density and osteoporotic fractures. Nat Commun. 2016;7:10129. 10.1038/ncomms10129 PMC4729819 26733130

[2] Hernlund E Svedbom A Ivergård M. Osteoporosis in the European Union: medical management, epidemiology and economic burden: a report prepared in collaboration with the International Osteoporosis Foundation (IOF) and the European Federation of Pharmaceutical Industry Associations (EFPIA). Arch Osteoporos. 2013;8:136. 10.1007/s11657-013-0136-1 PMC3880487 24113837

[3] Hegazy AA. Postponing menopause and lengthening fertile age for women’s good health: a potential hope. W J Gynecol Women’s Health. 2021;5:1–2.

[4] McPhee C Aninye IO Horan L. Recommendations for improving women’s bone health throughout the lifespan. J Womens Health (Larchmt). 2022;31:1671–6. 10.1089/jwh.2022.0361 PMC9805882 36346282

[5] Xiao PL Cui AY Hsu CJ. Global, regional prevalence, and risk factors of osteoporosis according to the World Health Organization diagnostic criteria: a systematic review and meta-analysis. Osteoporos Int. 2022;33:2137–53. 10.1007/s00198-022-06454-3 35687123

[6] Compston JE McClung MR Leslie WD. Osteoporosis. Lancet (London, England). 2019;393:364–76. 10.1016/S0140-6736(18)32112-3 30696576

[7] Svensson J Ohlsson C Karlsson MK. Higher serum free thyroxine levels are associated with increased risk of hip fractures in older men. J Bone Mineral Res. 2024;39:50–8. 10.1093/jbmr/zjad005 PMC11207919 38630877

[8] Attanasio R Žarković M Papini E. Patients’ persistent symptoms, clinician demographics and geo-economic factors are associated with choice of therapy for hypothyroidism by European thyroid specialists: the “THESIS” collaboration (Treatment of Hypothyroidism in Europe by Specialists, an International Survey). Thyroid. 2024;34:429–41. 10.1089/thy.2023.0580 38368541

[9] Delitala AP Scuteri A Doria C. Thyroid hormone diseases and osteoporosis. J Clin Med. 2020;9:1034. 10.3390/jcm9041034 PMC7230461 32268542

[10] Xie J Guo J Kanwal Z. Calcitonin and bone physiology: in vitro, in vivo, and clinical investigations. Int J Endocrinol. 2020;2020:3236828. 10.1155/2020/3236828 PMC7501564 32963524

[11] Caglar SE Karakoc Y Tanoglu A. Investigation of hemorheology in patients with hyperthyroidism via blood viscosity, erythrocyte deformability and aggregation. Thyroid Res. 2025;18:11. 10.1186/s13044-025-00227-w PMC11924651 40108642

[12] Yang J Ling X Yang Q Qiao Z. Fish bone penetrating into the thyroid gland: an unusual location of foreign body. Asian J Surg. 2024;47:4151–2. 10.1016/j.asjsur.2024.05.070 38772819

[13] Pörings AS Lowin T Dufner B Grifka J Straub RH. A thyroid hormone network exists in synovial fibroblasts of rheumatoid arthritis and osteoarthritis patients. Sci Rep. 2019;9:13235. 10.1038/s41598-019-49743-4 PMC6744488 31519956

[14] Wojcicka A Bassett JHD Williams GR. Mechanisms of action of thyroid hormones in the skeleton. Biochim Biophys Acta. 2013;1830:3979–86. 10.1016/j.bbagen.2012.05.005 22634735

[15] Opdebeeck B Van Den Branden A Adriaensen S. β,γ-Methylene-ATP and its metabolite medronic acid affect both arterial media calcification and bone mineralization in non-CKD and CKD rats. JBMR Plus. 2024;8:ziae057. 10.1093/jbmrpl/ziae057 PMC11102572 38764790

[16] Li C Hao J Wang C. Changes in drug clinical trials of thyroid diseases in China, 2009–2022. Drug Des Devel Ther. 2023;17:2315–24. 10.2147/DDDT.S409617 PMC10407876 37559911

[17] Badhey N Salvaji T Badhey H. Untreated primary hypothyroidism manifesting as cardiac tamponade. Cureus. 2024;16:e61169. 10.7759/cureus.61169 PMC11207166 38933628

[18] Tuchendler D Bolanowski M. Assessment of bone metabolism in premenopausal females with hyperthyroidism and hypothyroidism. Endokrynol Pol. 2013;64:40–4. 23450446

[19] Shim J Lin T Dashiell-Earp C Nechrebecki M Leung AM. Endocrinology practice patterns of hypothyroidism and osteoporosis management in a U.S. tertiary academic medical center. Clin Diabetes Endocrinol. 2019;5:10. 10.1186/s40842-019-0085-8 PMC6637534 31360539

[20] Dziedzic M Bonczar M Ostrowski P. Association between serum TSH concentration and bone mineral density: an umbrella review. Hormones. 2024;23:547–65. 10.1007/s42000-024-00555-w 38581565

[21] Deshmukh H Papageorgiou M Aye M England J Abdalla M Sathyapalan T. Hyperthyroidism and bone mineral density: dissecting the causal association with Mendelian randomization analysis. Clin Endocrinol (Oxf). 2021;94:119–27. 10.1111/cen.14330 32947644

[22] Sekula P Del Greco M F Pattaro C Köttgen A. Mendelian randomization as an approach to assess causality using observational data. J Am Soc Nephrol. 2016;27:3253–65. 10.1681/ASN.2016010098 PMC5084898 27486138

[23] Palmer TM Lawlor DA Harbord RM. Using multiple genetic variants as instrumental variables for modifiable risk factors. Stat Methods Med Res. 2012;21:223–42. 10.1177/0962280210394459 PMC3917707 21216802

[24] Bowden J Del Greco M F Minelli C Davey Smith G Sheehan N Thompson J. A framework for the investigation of pleiotropy in two-sample summary data Mendelian randomization. Stat Med. 2017;36:1783–802. 10.1002/sim.7221 PMC5434863 28114746

[25] Verbanck M Chen CY Neale B Do R. Publisher correction: detection of widespread horizontal pleiotropy in causal relationships inferred from Mendelian randomization between complex traits and diseases. Nat Genet. 2018;50:1196. 10.1038/s41588-018-0164-2 29967445

[26] Burgess S Thompson SG. Interpreting findings from Mendelian randomization using the MR-Egger method. Eur J Epidemiol. 2017;32:377–89. 10.1007/s10654-017-0255-x PMC5506233 28527048

[27] Hemani G Tilling K Davey Smith G. Correction: orienting the causal relationship between imprecisely measured traits using GWAS summary data. PLoS Genet. 2017;13:e1007149. 10.1371/journal.pgen.1007081 PMC5711033 29149188

[28] Williams GR Bassett JHD. Thyroid diseases and bone health. J Endocrinol Invest. 2018;41:99–109. 10.1007/s40618-017-0753-4 PMC5754375 28853052

[29] Bassett JHD Williams GR. Role of thyroid hormones in skeletal development and bone maintenance. Endocr Rev. 2016;37:135–87. 10.1210/er.2015-1106 PMC4823381 26862888

[30] Aubert CE Floriani C Bauer DC. Thyroid function tests in the reference range and fracture: individual participant analysis of prospective cohorts. J Clin Endocrinol Metab. 2017;102:2719–28. 10.1210/jc.2017-00294 PMC6283437 28482002

[31] Bjerkreim BA Hammerstad SS Eriksen EF. Bone turnover in relation to thyroid-stimulating hormone in hypothyroid patients on thyroid hormone substitution therapy. J Thyroid Res. 2022;2022:8950546. 10.1155/2022/8950546 PMC9553712 36248357

[32] Segna D Bauer DC Feller M. Association between subclinical thyroid dysfunction and change in bone mineral density in prospective cohorts. J Intern Med. 2018;283:56–72. 10.1111/joim.12688 PMC5739958 29034571

[33] Duncan Bassett JH Williams GR. The molecular actions of thyroid hormone in bone. Trends Endocrinol Metab. 2003;14:356–64. 10.1016/s1043-2760(03)00144-9 14516933

[34] Bassett JHD Williams GR. Critical role of the hypothalamic–pituitary–thyroid axis in bone. Bone. 2008;43:418–26. 10.1016/j.bone.2008.05.007 18585995

[35] Harvey CB O’Shea PJ Scott AJ. Molecular mechanisms of thyroid hormone effects on bone growth and function. Mol Genet Metab. 2002;75:17–30. 10.1006/mgme.2001.3268 11825060

[36] Lademann F Rijntjes E Köhrle J Tsourdi E Hofbauer LC Rauner M. Hyperthyroidism-driven bone loss depends on BMP receptor Bmpr1a expression in osteoblasts. Commun Biol. 2024;7:548. 10.1038/s42003-024-06227-0 PMC11078941 38719881

[37] Tuchendler D Bolanowski M. The influence of thyroid dysfunction on bone metabolism. Thyroid Res. 2014;7:12. 10.1186/s13044-014-0012-0 PMC4314789 25648501

[38] Sampath TK Simic P Sendak R. Thyroid-stimulating hormone restores bone volume, microarchitecture, and strength in aged ovariectomized rats. J Bone Miner Res. 2007;22:849–59. 10.1359/jbmr.070302 17352644

[39] Vestergaard P Rejnmark L Mosekilde L. Influence of hyper- and hypothyroidism, and the effects of treatment with antithyroid drugs and levothyroxine on fracture risk. Calcif Tissue Int. 2005;77:139–44. 10.1007/s00223-005-0068-x 16151671

[40] Xie J Xu J Chen H. Regulatory mechanisms of miR-212-3p on the secretion of inflammatory factors in monocyte–macrophages and the directed differentiation into osteoclasts in ankylosing spondylitis. Aging (Milano). 2023;15:13411–21. 10.18632/aging.205249 PMC10713416 38019469

[41] Vestergaard P Weeke J Hoeck HC. Fractures in patients with primary idiopathic hypothyroidism. Thyroid. 2000;10:335–40. 10.1089/thy.2000.10.335 10807062

[42] Ko YJ Kim JY Lee J. Levothyroxine dose and fracture risk according to the osteoporosis status in elderly women. J Prev Med Public Health. 2014;47:36–46. 10.3961/jpmph.2014.47.1.36 PMC3930806 24570805

[43] Kosińska A Syrenicz A Kosiński B Garanty-Bogacka B Syrenicz M Gromniak E. Osteoporosis in thyroid diseases. Endokrynol Pol. 2005;56:185–93. 16335687

[44] Stevens DA Harvey CB Scott AJ. Thyroid hormone activates fibroblast growth factor receptor-1 in bone. Mol Endocrinol. 2003;17:1751–66. 10.1210/me.2003-0137 12805413

