# Supplementary material for: Thyroid dysfunction and osteoporosis: A two-sample bidirectional Mendelian randomization study
Source: Medicine (Baltimore). 2025 Sep 26;104(39):e44779. doi: 10.1097/MD.0000000000044779 (PMC13593126; doi:10.1097/MD.0000000000044779)
Supplement: Supplementary file 1 [file medi-104-e44779-s001.pdf]

Table S1. Characteristics of the genetic variants associated with Hyperthyroidism

SNP	Chr	EA	Beta	SE	MAF	*P*-value	*F*-statistic*
rs10087240	8	T	0.001	0.0002	0.458	2.30E-10	39.860
rs112858426	6	G	0.008	0.0005	0.074	8.50E-65	297.125
rs11736377	4	T	-0.001	0.0002	0.263	1.40E-08	31.977
rs12067370	1	C	0.001	0.0002	0.335	1.10E-09	37.233
rs12721672	6	A	0.003	0.0003	0.121	1.70E-20	88.002
rs13250295	8	T	-0.001	0.0002	0.304	2.20E-09	35.713
rs141127771	6	A	0.003	0.0002	0.259	1.20E-42	188.527
rs147354869	6	G	-0.003	0.0004	0.081	4.70E-11	51.776
rs1559810	3	A	0.001	0.0002	0.406	2.20E-09	35.150
rs163315	5	T	0.001	0.0003	0.146	2.30E-08	31.537
rs185774696	6	T	0.003	0.0002	0.292	7.40E-39	169.431
rs200214283	6	T	0.004	0.0004	0.071	1.40E-27	123.519
rs200801362	6	C	0.005	0.0003	0.157	1.60E-50	232.205
rs2854001	6	A	-0.002	0.0002	0.224	6.90E-27	104.011
rs3087243	2	A	-0.002	0.0002	0.448	9.00E-27	114.841
rs3097674	6	C	0.002	0.0003	0.125	2.30E-10	41.594
rs376892	6	A	-0.001	0.0002	0.293	6.40E-11	44.706
rs41552812	6	T	0.008	0.0004	0.070	7.40E-77	350.985
rs4409785	11	C	0.001	0.0002	0.171	1.10E-09	36.895
rs538294600	6	A	-0.002	0.0002	0.367	1.30E-20	86.762
rs568609045	6	A	0.005	0.0004	0.088	3.60E-32	145.836
rs6679677	1	A	0.003	0.0003	0.097	1.60E-17	72.255
rs71536554	6	C	0.003	0.0003	0.169	1.70E-21	92.477
rs7747236	6	T	0.007	0.0003	0.132	4.80E-136	628.553
rs7749924	6	T	-0.002	0.0003	0.122	8.70E-12	58.500
rs80031348	6	A	0.004	0.0006	0.025	1.80E-12	49.907
rs80163833	6	T	-0.003	0.0002	0.270	3.40E-35	159.628
rs9265453	6	G	0.003	0.0002	0.399	1.20E-33	137.962
rs9393716	6	G	0.002	0.0002	0.195	1.30E-12	51.151

Table S2. Characteristics of the genetic variants associated with Hypothyroidism

SNP	Chr	EA	Beta	SE	MAF	*P*-value	*F*-statistic*
rs10036386	5	T	0.003	0.0004	0.386	3.50E-14	55.260
rs1003807	6	G	0.006	0.0006	0.156	1.20E-25	105.902
rs10073728	5	G	-0.003	0.0007	0.126	3.50E-08	28.938
rs10277273	7	G	-0.003	0.0004	0.428	4.60E-10	35.173
rs1032129	8	C	-0.003	0.0004	0.359	4.40E-12	46.228
rs10424978	19	A	-0.004	0.0004	0.394	2.30E-23	97.934
rs1045216	10	G	0.003	0.0004	0.402	1.80E-13	51.957
rs1050976	6	T	0.003	0.0004	0.483	1.30E-14	58.958
rs1064448	16	G	0.003	0.0004	0.484	4.10E-10	39.015
rs10748781	10	A	-0.003	0.0004	0.425	3.90E-12	47.967
rs10761620	10	G	-0.003	0.0004	0.454	2.00E-16	63.165
rs10772561	12	C	0.002	0.0004	0.373	4.60E-08	29.568
rs1079418	6	G	-0.003	0.0005	0.309	5.00E-11	42.836
rs10917470	1	A	0.003	0.0004	0.496	1.60E-10	38.055
rs10956412	8	C	-0.004	0.0006	0.169	7.30E-13	51.586
rs112165453	2	T	0.009	0.0016	0.019	1.40E-08	30.621
rs11231757	11	T	-0.003	0.0004	0.373	2.10E-12	45.736
rs11246217	11	G	-0.003	0.0005	0.276	4.70E-09	34.168
rs11256448	10	G	0.003	0.0005	0.246	4.00E-11	44.533
rs112923147	6	G	0.014	0.0007	0.225	7.69E-102	471.168
rs113229608	3	A	0.006	0.0009	0.063	3.20E-13	52.622
rs113473633	4	G	-0.010	0.0014	0.025	2.70E-11	46.943
rs114115115	6	G	-0.009	0.0009	0.061	3.60E-20	92.310
rs114378220	5	T	0.006	0.0009	0.071	5.00E-11	42.881
rs114473447	6	A	-0.011	0.0017	0.022	2.90E-11	40.145
rs115627609	6	T	0.013	0.0007	0.187	1.60E-83	385.276
rs115683811	6	G	-0.017	0.0021	0.011	7.90E-17	64.485
rs11582506	1	G	0.002	0.0004	0.492	3.90E-08	29.055
rs11666808	19	C	0.003	0.0004	0.370	1.40E-15	58.758
rs11675342	2	T	0.005	0.0004	0.423	1.20E-28	118.535
rs11711810	3	C	0.004	0.0006	0.129	2.10E-11	44.725
rs11783023	8	T	-0.003	0.0005	0.281	6.80E-10	35.950
rs12117927	1	A	0.003	0.0004	0.484	4.00E-10	36.493
rs12131057	1	A	-0.003	0.0005	0.255	9.30E-09	32.777
rs1217238	1	A	-0.003	0.0005	0.325	5.80E-10	37.252
rs12204380	6	C	0.003	0.0005	0.231	1.90E-08	32.884
rs12482947	21	C	0.003	0.0004	0.403	1.50E-10	38.884
rs1257926	14	A	0.003	0.0004	0.478	1.10E-10	40.484
rs12582330	12	T	-0.004	0.0005	0.283	1.20E-15	64.348
rs12634152	3	T	-0.006	0.0004	0.463	3.70E-52	224.826
rs12697352	5	A	-0.003	0.0005	0.334	3.10E-09	33.706
rs12742404	1	G	0.004	0.0007	0.118	1.30E-10	40.542
rs12796568	11	T	-0.005	0.0008	0.077	8.50E-09	33.639
rs13090803	3	T	0.005	0.0005	0.211	1.10E-18	77.139
rs13107325	4	T	-0.005	0.0008	0.072	2.70E-08	30.272
rs13259044	8	T	-0.003	0.0005	0.302	8.70E-13	48.407
rs13358767	5	G	-0.003	0.0005	0.279	2.20E-08	30.462
rs1345229	6	A	-0.005	0.0008	0.088	3.70E-09	39.752

rs138453996	16	A	0.010	0.0016	0.020	3.30E-11	44.159
rs138662522	6	C	-0.018	0.0027	0.012	1.30E-12	47.085
rs142647938	2	A	-0.010	0.0016	0.021	1.70E-10	38.305
rs142997491	16	G	0.013	0.0020	0.012	1.00E-10	40.017
rs145269503	6	A	-0.008	0.0015	0.020	8.50E-09	29.885
rs149941420	6	G	0.008	0.0009	0.092	4.80E-17	74.040
rs150332089	3	A	0.004	0.0006	0.137	1.00E-09	35.600
rs1534430	2	T	-0.004	0.0004	0.385	1.00E-18	75.464
rs1561924	8	A	-0.005	0.0006	0.132	9.30E-13	51.971
rs17020139	1	A	0.011	0.0008	0.090	2.50E-51	222.261
rs1723022	1	T	0.003	0.0005	0.363	8.60E-09	32.011
rs1810396	8	G	-0.004	0.0005	0.306	1.10E-17	73.868
rs1884352	1	A	-0.002	0.0004	0.384	9.50E-09	31.947
rs200214283	6	T	0.007	0.0009	0.071	7.40E-13	56.782
rs200545029	11	C	-0.006	0.0009	0.058	7.10E-13	48.775
rs2111485	2	G	0.004	0.0004	0.406	2.50E-18	75.667
rs212411	6	A	0.002	0.0004	0.441	2.00E-08	30.190
rs2155829	8	C	0.003	0.0004	0.392	3.40E-11	42.315
rs221781	7	G	0.005	0.0007	0.113	9.70E-12	44.255
rs2234167	1	A	0.004	0.0006	0.133	3.20E-11	42.263
rs2248605	13	G	-0.003	0.0005	0.268	6.30E-09	31.490
rs229540	22	G	0.005	0.0004	0.432	2.80E-33	145.533
rs2445610	8	G	-0.004	0.0004	0.367	2.90E-16	66.244
rs244690	5	A	-0.005	0.0006	0.136	1.90E-13	56.807
rs2452170	19	A	0.002	0.0004	0.478	2.20E-08	31.508
rs2473808	1	C	-0.003	0.0005	0.334	8.50E-12	46.246
rs2476601	1	G	-0.020	0.0007	0.097	4.80E-178	795.790
rs2607013	6	T	0.007	0.0006	0.181	1.70E-34	161.686
rs28157	5	T	-0.003	0.0005	0.308	1.10E-12	50.556
rs28375404	6	T	-0.011	0.0011	0.039	4.40E-24	96.680
rs28594633	6	A	0.011	0.0005	0.344	3.10E-86	396.842
rs28780083	6	G	-0.006	0.0007	0.112	1.70E-12	69.444
rs3087243	2	A	-0.008	0.0004	0.448	1.60E-86	378.211
rs3093665	6	C	0.010	0.0015	0.021	2.50E-11	46.867
rs31216	5	T	0.003	0.0006	0.130	2.00E-08	28.587
rs3184504	12	C	-0.010	0.0004	0.464	4.60E-124	550.391
rs34132030	13	T	0.004	0.0005	0.290	2.00E-14	57.899
rs34477738	9	G	0.004	0.0006	0.160	6.70E-12	48.604
rs35074907	19	A	0.009	0.0015	0.020	5.30E-10	36.727
rs35407628	13	T	-0.003	0.0005	0.201	1.10E-09	34.717
rs367023	2	G	-0.003	0.0005	0.298	2.80E-09	35.824
rs3775291	4	T	-0.004	0.0005	0.292	5.00E-18	73.953
rs3807307	7	C	0.003	0.0004	0.460	2.70E-10	39.153
rs3847800	12	G	0.003	0.0006	0.165	1.40E-08	33.568
rs3996020	3	A	0.003	0.0005	0.311	2.00E-10	40.328
rs41555717	6	G	-0.004	0.0006	0.181	6.00E-13	50.868
rs4320727	1	A	0.003	0.0004	0.380	6.90E-11	42.472
rs4409785	11	C	0.007	0.0006	0.171	3.00E-32	134.330
rs4444866	4	T	-0.003	0.0005	0.270	5.40E-12	47.548
rs4647211	3	C	-0.002	0.0004	0.445	3.90E-08	32.475

rs4804413	19	T	0.003	0.0004	0.421	2.20E-08	35.317
rs4820437	22	C	-0.003	0.0005	0.241	5.70E-09	33.742
rs4824117	22	G	-0.003	0.0005	0.336	4.50E-08	32.412
rs484959	1	C	0.003	0.0004	0.459	7.70E-11	42.265
rs509420	1	G	-0.003	0.0004	0.450	2.80E-09	33.609
rs535850137	12	A	0.004	0.0005	0.405	4.00E-14	56.078
rs543135444	17	T	0.005	0.0006	0.153	7.10E-16	66.660
rs555911977	6	T	0.011	0.0006	0.276	1.60E-73	342.687
rs557444314	1	C	0.010	0.0019	0.019	1.80E-08	30.215
rs56249713	18	C	-0.003	0.0004	0.412	3.30E-11	40.505
rs570623084	6	G	-0.011	0.0018	0.029	1.40E-09	40.055
rs5763767	22	A	-0.003	0.0004	0.454	9.60E-12	45.470
rs5865	2	T	-0.003	0.0005	0.347	1.70E-09	36.658
rs60115988	18	A	0.003	0.0006	0.167	1.90E-09	36.096
rs60600003	7	G	0.004	0.0007	0.098	2.80E-10	38.095
rs61759532	17	T	0.004	0.0005	0.238	8.70E-15	57.664
rs61766480	1	C	0.003	0.0005	0.469	3.90E-08	31.515
rs61776678	1	A	-0.003	0.0004	0.405	8.50E-10	36.429
rs61983206	14	T	-0.004	0.0005	0.464	6.60E-14	55.107
rs62261536	3	C	-0.004	0.0006	0.138	5.80E-10	35.883
rs654537	6	A	0.006	0.0004	0.383	3.00E-41	179.019
rs6815267	4	C	-0.006	0.0005	0.217	2.00E-33	142.570
rs6833591	4	G	-0.003	0.0005	0.347	5.70E-11	43.272
rs7090530	10	A	0.004	0.0004	0.397	1.40E-19	81.943
rs7134472	12	A	-0.002	0.0004	0.380	2.90E-08	30.053
rs71377264	17	A	0.005	0.0006	0.151	4.10E-14	57.353
rs71508903	10	T	0.007	0.0006	0.192	2.00E-35	150.844
rs71571466	6	A	0.005	0.0009	0.061	8.50E-09	31.826
rs72678531	1	C	0.003	0.0006	0.165	1.60E-09	32.764
rs7282047	21	G	-0.003	0.0005	0.321	1.30E-12	49.551
rs73191857	12	T	-0.005	0.0009	0.062	1.90E-08	28.664
rs736374	11	A	0.004	0.0004	0.361	7.50E-19	78.354
rs7417283	1	T	0.004	0.0005	0.195	8.70E-12	46.144
rs7441808	4	G	0.004	0.0005	0.292	2.60E-18	70.686
rs744254	10	A	0.003	0.0005	0.274	3.20E-12	49.778
rs75589097	6	C	0.014	0.0019	0.017	7.20E-13	55.271
rs7568273	2	G	0.003	0.0005	0.435	2.70E-09	34.671
rs7583027	2	C	0.003	0.0005	0.352	4.80E-11	45.082
rs7596240	2	G	0.003	0.0005	0.268	8.10E-09	32.179
rs76428106	13	C	0.026	0.0020	0.013	1.30E-38	168.264
rs76930710	12	C	-0.006	0.0011	0.038	1.40E-08	29.491
rs7705526	5	A	-0.003	0.0005	0.326	6.80E-10	39.359
rs7762909	6	G	0.006	0.0005	0.334	6.00E-42	167.762
rs78041260	3	A	0.004	0.0007	0.118	3.50E-08	28.973
rs7849424	9	C	0.003	0.0004	0.395	1.20E-08	33.389
rs7850258	9	G	0.010	0.0005	0.326	1.10E-109	482.511
rs7937786	11	A	-0.002	0.0004	0.407	2.20E-08	30.734
rs79708723	6	A	0.007	0.0012	0.033	1.00E-08	33.657
rs8008961	14	T	-0.003	0.0005	0.280	2.30E-11	41.556
rs8043085	15	T	0.004	0.0005	0.235	1.10E-16	63.153

rs8054578	16	G	-0.003	0.0005	0.233	1.70E-10	40.064
rs926103	1	C	-0.003	0.0005	0.354	1.10E-08	32.141
rs9262551	6	C	0.013	0.0015	0.020	6.20E-16	70.652
rs9272426	6	G	0.012	0.0004	0.449	4.30E-163	765.681
rs9277398	6	C	-0.008	0.0005	0.313	3.30E-61	271.001
rs9347170	6	T	-0.005	0.0005	0.349	7.00E-34	144.252
rs9357119	6	T	-0.009	0.0005	0.282	1.10E-85	329.388
rs9380522	6	T	0.003	0.0005	0.290	1.30E-10	42.062
rs9497965	6	T	0.004	0.0004	0.409	2.50E-18	76.438
rs9507287	13	C	-0.006	0.0005	0.336	9.50E-41	179.803
rs9568389	13	A	0.004	0.0008	0.086	3.00E-08	31.736
rs9619183	22	T	-0.005	0.0009	0.058	3.60E-08	31.075
rs9697210	9	A	-0.004	0.0006	0.144	1.60E-09	37.100
rs970987	9	A	-0.004	0.0005	0.347	1.50E-17	71.010
rs9831283	3	A	0.006	0.0010	0.044	3.30E-09	33.551

Table S3. Characteristics of the genetic variants associated with Osteoporosis.

SNP	Chr	EA	Beta	SE	MAF	*P*-value	*F*-statistic*
rs10023907	4	T	0.002	0.0003	0.338	4.30E-12	47.545
rs10280461	7	A	-0.002	0.0003	0.461	6.00E-14	57.089
rs10281460	7	G	-0.002	0.0003	0.231	4.00E-08	29.619
rs10490823	3	T	-0.002	0.0003	0.462	6.80E-12	46.394
rs11802513	1	A	-0.002	0.0003	0.180	9.90E-12	46.224
rs118115924	12	T	0.007	0.0012	0.011	2.20E-08	30.811
rs12044944	1	T	-0.002	0.0003	0.193	5.40E-09	33.942
rs188810925	17	A	-0.003	0.0005	0.076	3.50E-10	38.817
rs1936806	6	T	-0.001	0.0003	0.444	6.50E-09	33.606
rs2566755	1	C	-0.002	0.0003	0.218	1.30E-12	50.152
rs34325313	20	C	0.002	0.0004	0.125	5.50E-09	33.244
rs3801387	7	G	-0.002	0.0003	0.278	2.20E-17	70.998
rs56312530	11	A	0.002	0.0003	0.166	1.50E-10	40.928
rs8095551	18	C	-0.002	0.0003	0.236	2.10E-08	31.136
rs851974	6	A	0.001	0.0003	0.423	1.40E-08	32.354
rs9594738	13	T	0.002	0.0003	0.476	8.70E-16	64.799
rs9982895	21	C	0.002	0.0003	0.277	2.10E-08	31.813
